# Phase Transition in Iron Thin Films Containing Coherent Twin Boundaries: A Molecular Dynamics Approach

**DOI:** 10.3390/ma13163631

**Published:** 2020-08-17

**Authors:** Binjun Wang, Yunqiang Jiang, Chun Xu

**Affiliations:** School of Material Science and Engineering, Shanghai Institute of Technology, Haiquan Road 100, Shanghai 201418, China; 186082110@mail.sit.edu.cn

**Keywords:** solid–solid phase transition, iron thin film, free surface, twin boundary, molecular dynamics simulation

## Abstract

Using molecular dynamics (MD) simulation, the austenitic and martensitic phase transitions in pure iron (Fe) thin films containing coherent twin boundaries (TBs) have been studied. Twelve thin films with various crystalline structures, thicknesses and TB fractions were investigated to study the roles of the free surface and TB in the phase transition. In the austenitic phase transition, the new phase nucleates mainly at the (112)_bcc_ TB in the thicker films. The ($11\bar{1}$)_bcc_ free surface only attends to the nucleation, when the film is extremely thin. The austenitic transition temperature shows weak dependence on the film thickness in thicker films, while an obvious transition temperature decrease is found in a thinner film. TB fraction has only slight influence on the austenitic temperature. In the martensitic phase transition, both the ($\bar{1}10)$_fcc_ free surface and (111)_fcc_ TB attribute to the new body-center-cubic (bcc) phase nucleation. The martensitic transition temperature increases with decreased film thickness and TB fraction does not influent the transition temperature. In addition, the transition pathways were analyzed. The austenitic transition obeys the Burgers pathway while both the Kurdjumov–Sachs (K–S) and Nishiyama–Wassermann (N–W) relationship are observed in the martensitic phase transition. This work may help to understand the mechanism of phase transition in the Fe nanoscaled system containing a pre-existing defect.

## 1. Introduction

Due to the industrial and scientific importance of the solid–solid phase transition, in particular the α/γ phase transition in pure iron (Fe) or in steel, significant efforts have been made to reveal the transition mechanisms [1,2,3,4]. In the recent past, interest is rising in studying the solid–solid phase transition in nanoscaled or surface dominated Fe-systems, because phase transition caused structural changing is directly associated with many of the unique physical and magnetic properties of Fe [5,6]. Compared to the bulk materials, such systems exhibit different features in phase transition due to their high surface–volume ration, for instance, surface nucleation could be often observed and some interesting phenomena such as structural reorientation or back transition to the original phase at high tensile strains in strain induced phase transition have been reported [7,8,9,10,11]. In these works, phase transitions were often discussed in dependence on surface fraction and orientation. More recently, Meiser and Urbassek [12] studied systematically the influence of different orientated face-center-cubic (fcc) and bcc surfaces on the austenitic and martensitic phase transitions using molecular dynamics (MD) simulation. They found that the some surfaces, for instance, the (111)_bcc_ surface, do not assist the phase transition as nucleation sites, while most of the free surfaces serve as the sites of the new phase nucleation.

In the bulk material, crystal defects influent the transition behavior of Fe dramatically. Grain boundaries (GBs) [13], twin boundaries (TBs) [14] or dislocations [15,16,17] often act as the nucleation sites of the new phase. However, the influence of crystal defects on the transition in nanoscaled systems is still under discussion. Recently, it has been reported that the new phase prefers to nucleate at dislocation sites, in spite of the existence of the free surface in Fe thin slabs [17]. Here, the different defect structures, such as dislocations, stacking faults (SFs) and TBs, are important as they are present in the material in reality, depending on the alloy composition and applied heat or mechanical treatment [3,4,18]. In addition, the energetics of such defects is also distinct in Fe [14,19,20]. Thus, the transition mechanism may be differently affected by the crystal defects with different energetics and structures.

Among these crystal defects, TB provides one example that dramatically influents the mechanical properties, for instance, the twinning induced plasticity (TWIP) steel [21,22], and the transition behavior [4,14]. However, most of the studies focused on the effect of deformation twin on the transition behavior [4,23]. Another type of TB, the annealing twin or growth twin, is also present in Fe [24,25,26], which may also have effect on the phase transition, especially in the nanoscaled system such as the Fe thin film. The present work aims to observe the austenitic and martensitic phase transitions during heating/cooling in the presence of free surfaces and TBs in the parent phase. The modeled TBs will be structurally and energetically analyzed. We aim to reveal the roles of free surface and TB as nucleation sites on the austenitic and martensitic phase transitions and the possible competition between them. Furthermore, the influence of surface and TB fraction on the phase transitions will be studied. Finally, the transition pathways will be analyzed. This work may help to understand the mechanism of phase transition in Fe nanoscaled system containing pre-existing defects.

## 2. Simulation Methodology

The choice of interatomic potential plays a critical role in MD simulation. For Fe, Engin et al. [27] analyzed six embedded atom method (EAM) potentials and concluded that among them, only the Meyer–Entel potential [28] is able to describe both the austenitic and martensitic phase transitions. The Meyer–Entel potential predicts a lattice constant of the bcc (fcc) phase of 2.869 Å (3.686 Å) at 300 K, whereas the lattice constant of the bcc (fcc) phase is experimentally found to be 2.866 Å (3.571 Å) [29]. This slightly higher atomic volume of the fcc phase causes incorrect atomic volume relationship during the phase transitions. A volume increase was observed during the austenitic transition and a volume decrease was observed during the martensitic transition [30], which is opposite to the experimental observation. However, we should note that this incorrect relationship only resolves the signs of the stresses during the austenitic/martensitic phase transition. Assuming the Bain path of martensitic phase transition, in which two axes of the bcc lattice should be expanded and one axis should be compressed, the Meyer–Entel potential gave two compressive stresses and one tensile stress [30]. We note that these stresses induced by the phase transitions are not high, which are smaller than 0.1 GPa (0.6 GPa) for all the austenitic (martensitic) transitions in the present work and may not have big influence on the transition mechanism. Song et al. [31] showed that this opposite trend in studying the bcc phase nucleation at GBs and pointed out that this discrepancy does not affect the nucleation and growth of the bcc phase. We list here some data to illustrate the quality of the Meyer-Entel potential. Except the lattice constants mentioned above, the Meyer–Entel potential predicts the elastic constants C_11_, C_12_ and C_44_ of 1.528, 0.784 and 0.702 eV/Å^3^ [27], which differ only slightly from the experimental values [32]. We calculated the constant-volume heat capacity (C_v_) of the bcc phase and obtained a value of 3144.8 kJ/(m^3^·K) (experimental value 3534 kJ/(m^3^·K)). Most importantly, the Meyer-Entel potential provides correct energetics of the planar defects, which is involved in this work. Wang and Urbassek [33] calculated the {111}, {110} and {001} surface energies of both bcc and fcc phases using the Meyer-Entel potential and the values basically correlate with the experimental and ab initio data [34,35,36,37]. The authors also calculated the phase boundary energy in a Nishiyama-Wassermann (N-W) relationship and found the result agrees satisfactorily with the experimental value [38]. More recently, Karewar et al. [14] calculated the SF energy of fcc phase along the {111} <112> slip system using the Meyer-Entel potential and a negative value of −54 mJ/m^2^ was reported, while a positive value of 37.5 mJ/m^2^ was found using a modified embedded atom model (MEAM) potential developed by Lee et al. [39], which may be also able to represent both the austenitic and martensitic phase transitions. Karewar et al. [14] emphasized the correctness of this negative value and pointed out that it indicates a spontaneous formation of the planar defects in the fcc phase. Two first principles studies on the fcc Fe reported that the SF energy of non-magnetic fcc Fe is −415 mJ/m^2^, while the value of paramagnetic fcc Fe is −105 mJ/m^2^ [40,41]. The SFE given by the Meyer-Entel potential is in satisfied agreement with the paramagnetic fcc phase, as in reality, although the magnetic effect was not considered in the Meyer-Entel potential due to its EAM formalism, discussion about the effect of magnetic effect on equilibrium transition temperature see below. By comparing the SFE given by the Meyer-Entel potential and the first principles data mentioned above, the authors of reference [14] did not use the Lee potential in their work. We calculated the {112}_fcc_ twin boundary energy (SFE) and it is −7.3 mJ/m^2^, for details see Section 3.1. It can be concluded that the Meyer-Entel potential correctly represents the basic material properties. Finally, we discuss the equilibrium α/γ transition temperature of the Meyer-Entel potential. The free energy vs. temperature curves of the bcc and fcc phases intersect at a temperature of 550 ± 50 K [27], which differs from the experimentally measured value of 1184 K. This is because this potential does not consider the magnetic entropy contribution, which stabilizes the bcc phase at low temperatures [14,25,42,43]. However, the transition mechanism could be assumed not to be influenced [1,14]. We notice that another bond order potential developed by Müller et al. [44] predicts an α/γ transition temperature of 1030 K in the Fe bulk system, which is much closer to the reality. The authors pointed out that their potential might be not suitable to describe the finite-size effect. However, we still performed simulations using this potential and no austenitic and martensitic phase transitions could be observed during heating/cooling in Fe thin films. Note that a single simulation lasted over 33 h for a system containing 80,000 atoms using the Müller potential on a platform with 64 CPU cores (2.2 GHz), while the Meyer-Entel potential provided a total simulation time of less than two hours. Considering these literature studies, the relative precise basic material properties and the correct energetic of the planar defects, the Meyer-Entel potential may be reliable to describe the qualitative transition behavior. Thus, we chose this potential for our simulations. The performances of the potentials discussed above are briefly summarized by an additional table in the Appendix A.

To study the influence of film thickness and TB fraction on the austenitic phase transition, six thin films in the bcc structure were constructed. Three of them (films 1, 2 and 3) contained four (112)_bcc_ TBs, which are perpendicular to the ($11\bar{1}$)_bcc_ surfaces. These films vary in thickness and will be studied in Section 3.2.1. The other three (films 4, 5 and 6) have the same crystalline orientation as the first three. They have the same thickness, but vary in the number of (112)_bcc_ TBs contained, and will be studied in Section 3.2.2. Film 4 is a single crystal as reference. Detailed information of films 1–6 is listed in Table 1.

In order to investigate the influence of the film thickness and the TB fraction on the martensitic phase transition, another six thin films were constructed (films 7–12) in the fcc structure. Analogous to the bcc films, film thickness was varied in films 7–9, in which the (111)_fcc_ TBs were perpendicular to the ($\bar{1}10$)_fcc_ free surface. The orientations of films 10–12 were equivalent to those of films 7–12. Films 10–12 had the same thickness but varied in the number of TBs contained. Film 10 was a single crystal fcc film. The specifications of films 7–12 are listed in Table 2. Films 7–9 and 10–12 will be studied in Section 3.2.1 and Section 3.2.2, respectively.

**Figure 1:**
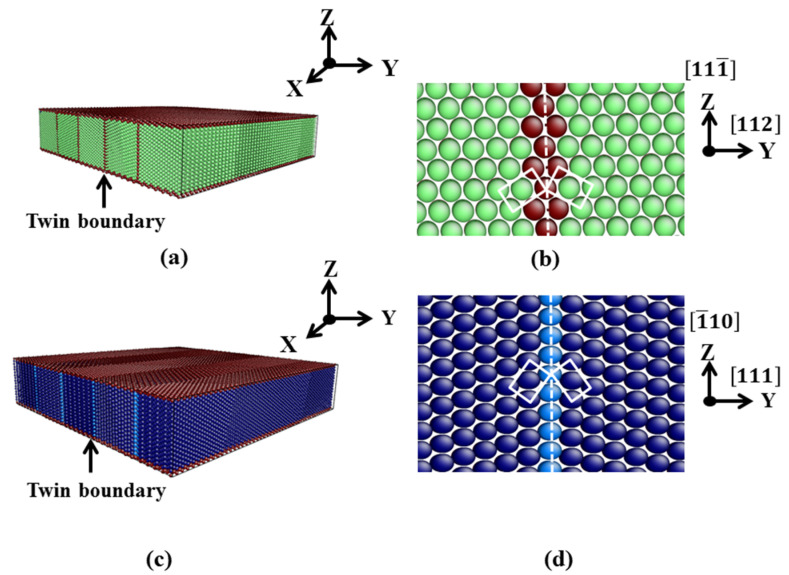
Setup of the thin films in the simulation. Colors denote the local crystal structure: green, bcc; dark blue, fcc; light blue, hcp; red, unknown. (**a**) Film 2 as an example for the bcc films; (**b**) structure for the constructed (112)_bcc_ TB; (**c**) film 8 as an example for the fcc films; and (**d**) structure for the constructed (111)_fcc_ TB. In (**b**) and (**d**), the white dashed lines and the white rectangles indicate the TBs and the mirrored unit cells.

Film 2 is taken as an example to describe our modeling. A simulation box with the dimensions of 202.9 Å × 42.2 Å × 89.4 Å was constructed and filled with Fe atoms in the bcc structure. The orientations of the bcc crystallite are depicted in Table 1. Then, a mirror symmetry operation of the simulation box with bcc atoms was performed with respect to the (112) plane that was positioned at y = 0. The mirrored crystal was placed directly beside the initial one. By repeating this operation another three times, the final structure of film 2 was obtained. The constructed TB was identified as a single layer of hexagonal-close-packed (hcp) atoms in the fcc structure, see Figure 1c,d. The other films were modeled in a similar manner.

The (112)_bcc_ and (111)_fcc_ twin boundary energy (TBE) were calculated using the following equation:

(1) $$\mathrm{TBE}=\frac{E_{\mathrm{twinning}}-E_{\mathrm{single}}}{A}$$

where E_twinning_ is the equilibrium energy the film containing TBs and E_single_ is the equilibrium energy of a single crystal film with the exactly same dimension, surface orientation and atom number as the film containing TBs. A is the total area of the TBs. The equilibrium energies were calculated using the energy minimizations by conjugate gradients. The tolerance of the energy minimization was set very strictly. Only when the energy difference between two successive steps divided by the energy magnitude smaller than 10^−30^, the energy minimization will be stopped.

For all films, periodic boundary conditions were employed in the x and y directions. In the z direction, which corresponds to the surface normal, the film surfaces were free. The films were equilibrated in an anisotropic NPT ensemble at a temperature of 50 K for the bcc films 1–6 and 600 K for the fcc films 7–12 using a Nóse-Hoover thermostat. Here we should note again that the Meyer-Entel potential predicts the α-γ transition temperature of 550 ± 50 K, as determined by the free energy calculations [27]. The pressure control is set to 0 in the x and y directions. No pressure control is employed in the z direction. The whole equilibration time for each film amounts to 50 ps. After equilibration, the pressures were smaller than 10 MPa in the lateral directions and the pressure in the z direction relaxed automatically to 0 due to the free surface. We should note that no TB–TB interaction and dislocation generation were observed during the equilibration, so only the free surfaces and TBs had influence on the phase transition in the afterward simulations.

After equilibration, our simulations are performed in an anisotropic NPT ensemble. The temperature was increased from 50 to 1500 K with a heating rate of 1 K/ps for the bcc films 1–6 and decreased from 600 to 10 K with a cooling rate of 0.33 K/ps for the fcc films 7–12. Martensitic phase transitions during cooling with a rate of 1 K/ps were not observed. It is well known that the heating/cooling rate has big influence on the austenitic/martensitic transition temperature [45]. In a previous work using the Meyer-Entel potential [30], it was reported that the austenitic temperature increases and the martensitic temperature decreases with a heating/cooling rate increase. Meiser and Urbassek [12] reported that a bulk fcc single crystal does not undergo a martensitic transition during cooling with a rate of 0.33 K/ps. In addition, the authors also simulated a thin fcc slab with the (110)_fcc_ free surface, like our films 7–12. A very low transition temperature of 81 K during cooling with a rate of 0.33 K/ps was reported. However, the martensitic phase transition starts in the bulk material rather than the free surface. The authors contributed this to the reality that the (110)_fcc_ surface is not conserved under any fcc/bcc orientation relationships. However, in references [14,30], martensitic phase transitions could be achieved at temperatures around 280 K with a cooling rate of 1 K/ps, even periodic boundary conditions were employed in all directions. We notice that the starting points of the transitions in references [14,30] were fcc crystals, which were transformed from bcc phases. In these works, heating/cooling cycles were applied to the systems. During the heating, the atoms leave their equilibrium positions due to the strong thermal vibration. In another work using the Meyer-Entel potential, over 40% of the atoms could not be identified at high temperatures [33], i.e., the system at high temperatures may contain rich varieties of imperfections. The atoms may not migrate back to their equilibrium positions due to the high cooling rate in the MD simulation. Besides TB and SF [14,33], vacancies should be remained in the system, for instance, at the TB or SF. It has been shown that the vacancies help the new phase nucleation because they introduce free volume in the material, which benefits the atom movement during the phase transition [28]. Note that in our work and reference [12], the starting points of the martensitic phase transitions were directly modeled fcc crystals only containing the surface and TB (or only surface). Thus, the starting points should be the reason for the reality that the martensitic phase transitions could not be achieved by using a cooling rate of 1 K/ps and the low martensitic transition temperatures in Section 3.3.1 and Section 3.3.2. Finally, we defined the austenitic (martensitic) transition temperature as the temperature, at which 50% of the bcc (fcc) atoms are transformed, in the present work.

The films were constructed using the program ATOMSK [46]. We used the common neighbor analysis (CNA) [47] to determine the local atomic structure. The dislocation extraction algorithm (DXA) [48], which is integrated in the visualization software OVITO [49], was used to identify the possible TB dislocations. The visualization was realized by using the ATOMEYE [50] code. All the simulations were performed with the open source LAMMPS code [51].

## 3. Results

### 3.1. TB in the bcc and fcc Structure

After equilibration, the (112)_bcc_ and (111)_fcc_ TBs maintained their initial structures. The DXA analysis did not indicate any twinning dislocations at the TBs and no twin–surface or twin–twin interactions were found. This is normal, since the most Fe interatomic potentials can represent stable coherent TBs in both bcc and fcc structures [19,52].

**Figure 2:**
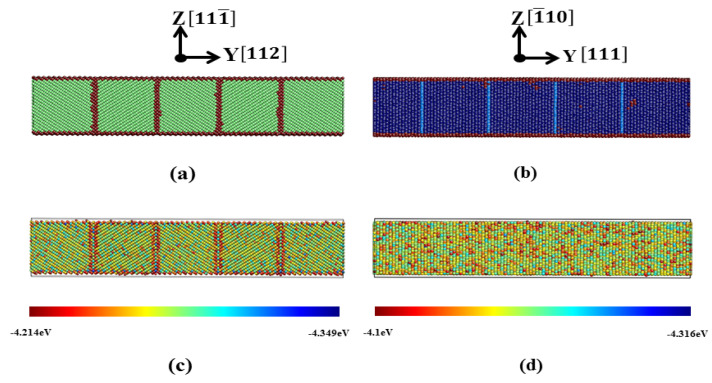
Statuses after energy minimization. (**a**) The (1$\bar{1}$0)_bcc_ plane of film 2; (**b**) the (11$\bar{2}$)_fcc_ plane of film 8. Colors denote the local structure as in Figure 1. (**c**) Potential energy distribution of the (1$\bar{1}$0)_bcc_ plane of film 2 and (**d**) potential energy distribution of the (11$\bar{2}$)_fcc_ plane of film 8. Atoms are colored according to their potential energy, see color bar.

Figure 2 shows the ($1\bar{1}0$)_bcc_ plane of film 2 (a and c) and (11$\bar{2}$)_fcc_ plane of film 8 (b and d) after equilibration. In Figure 2c,d, the surface atoms with obviously higher potential energies (around −3.3 eV for the atoms on the first layer at the ($11\bar{1}$)_bcc_ and ($\bar{1}10$)_fcc_ surfaces) are not shown. The equllibrium energy of a single bcc (fcc) atom was around −4.27 eV (−4.24 eV) predicted by the Meyer-Entel potential at 0 K [10,27]. The potential energies of the atoms at the TBs in the bcc film were slightly higher than that of the bulk atoms, while the TB atoms did not show obvious increased potential energies in the fcc film, c.f., Figure 2c,d. This indicates that the {111}_fcc_ TB was energetically more stable than the {112}_bcc_ TB.

Our calculated {112}_bcc_ and {111}_fcc_ TBEs amount to 0.176 J/m^2^ and −0.0073 J/m^2^ at 0 K. Papon et al. [20] calculated the {112}_bcc_ Fe TBE from a tight-binding description of the attractive d band and obtained a value of 0.171 J/m^2^, which is in fairly good agreement with our result. More recently, the {112}_bcc_ TBE has been calculated by Shituba et al. [19] using the Finnis–Sinclair (FS) potential [53] and their value was 0.6 J/m^2^. The authors also calculated the {111}_fcc_ TBE and reported it is almost 0. This result is in satisfactory agreement with our −0.0073 J/m^2^, which indicates the high stability of the constructed {111}_fcc_ TBs. Note that {112}_bcc_ TBs have higher energy than {111}_fcc_ TBs. This is due to the different atomic arrangement of the TBs in fcc and bcc structures. The stacking interruption caused by the (111)_fcc_ TB consitis only of one atom layer, as shown in Figure 2b. In contrast, the exsitence of (112)_bcc_ TB interrupts the stacking sequence of several atom layers [54]. This explains why the {111}_fcc_ TBE is close to zero and the {112}_bcc_ TBE exhibits a finite value.

Our calculated TBEs should be compared with the surface energies, since free surfaces and TBs both exist in our films. According to a previous study [33], the Meyer-Entel potential predicts the {111}_bcc_ and {110}_fcc_ surface energies of 1.844 J/m^2^ and 1.663 J/m^2^ at 0 K. Note that these two surfaces correspond to the surfaces of our simulated films, see Table 1. It is clear that our calculated TBEs were much lower than the surface energies. It can be concluded TBs in our simulated films were energetically more stable than free surfaces.

### 3.2. Austenitic Transition

#### 3.2.1. Dependence on Film Thickness

**Figure 3:**
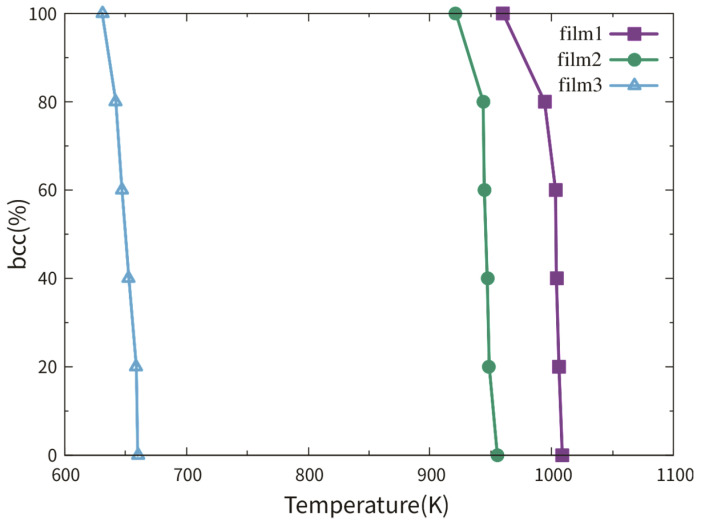
Fractional bcc phase content of films 1–3 as a function of temperature. Film 3 is half as thick as film 2 and 1/4 as thick as film 1. Each film contains 4 TBs.

We first discuss the austenitic temperature dependence on the film thickness via films 1–3. Figure 3 shows the fractional bcc phase content as a function of temperature. The austenitic transition temperatures of films 1–3 were around 1005 K, 945 K and 650 K, respectively. It seems that the austenitic temperature decreased with the thickness decrease. This tendency has been also reported in a previous work studying the transition temperature dependence on the thickness in Fe thin slabs [12]. The authors explained this tendency by the higher surface fraction in thinner films, which provides more space for the new phase nucleation. However, the austenitic transition temperatures of films 1 and 2 differed only by 60 K, while the transition temperature difference between films 2 and 3 was 295 K.

**Figure 4:**
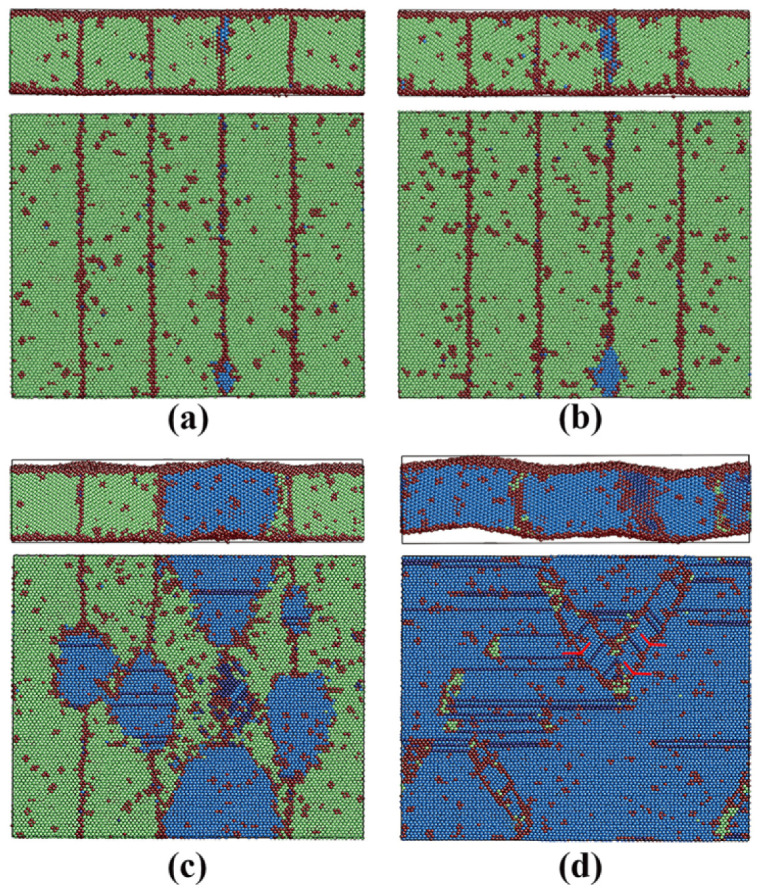
Snapshots of film 2 during the austenitic phase transition. Colors denote the local crystal structure as in Figure 1. In a–d, the upper figures show the side view in the [$1\bar{1}0$]_bcc_ direction (x) and the lower figures show the vertical views in the [$11\bar{1}$]_bcc_ direction (z). In the lower figures, the film is cut in the middle along the ($11\bar{1}$)_bcc_ plane and the upper part is removed. (**a**) The first nucleation; (**b**) growth of the new phase along the TB; (**c**) new nucleation and afterward growth; and (**d**) final status after the transition (Figure 4a,b have been revised.).

To clarify this, the local atomic structure during the austenitic transition was analyzed. The transition processes of films 1 and 2 were similar. Figure 4 displays the atomic snapshots of film 2 during the austenitic transition. The new phase nucleates firstly at the TB, or more exactly, at the cross position between the free surface and the TB, as shown in Figure 4a. This is not unexpected because this position provides the biggest energy and structure fluctuations, which are necessary for the new phase nucleation. The new phase, which is identified as hcp, propagates firstly along the TB until the whole TB is transformed, as shown in Figure 4b. It should be explained why the new phase is in the hcp structure rather than fcc. It might be possible that the system is not sufficiently equilibrated and the residual stresses remained in the film may induce the bcc→hcp transition. Our equilibration process was checked again. Due to the barostate and the free surfaces, the stresses in all three directions are well controlled/relaxed at 0 in the middle and last stage of the equilibration. Thus, the stress induced bcc→hcp phase transition can be excluded. We note that such bcc→hcp phase transitions have been reported in MD simulations using the Meyer-Entel potential a few times, not only in stress/strain induced transitions [7,10,11], but also in transitions induced by temperature increasing [8,33,55]. This bcc→hcp transition should be discussed from two aspects. Firstly, the free energy difference between the fcc and hcp phases amounts to 4 meV/atom predicted by the Meyer-Entel potential [42]. Secondly, the transition barriers between the fcc and hcp phases were calculated, which were 9.6 meV/atom from hcp to fcc and 5.6 meV/atom from fcc to hcp. These values were tiny, indicating that very small temperature or pressure fluctuations may cause mutual transformation between the two phases. Since the fcc and hcp phase both exhibited a close packed (cp) structure, it is hence more appropriate to denote the austenitic transition as a bcc→cp transformation. As the transformed TB grew in the transverse directions, c.f., the upper Figure 4c,d, some new hcp phases also nucleated at other TBs, as shown in the lower Figure 4c. The cp phases nucleated at all the TBs had roughly equivalent orientation and combined with each other after growth, as shown in Figure 4d. The whole system transformed from its original bcc structure into the cp structure (mainly hcp). Only several grains resulted, of which one dominated the film volume. The small grains were separated from the dominated one with TBs, which are indicated by the red lines in the lower Figure 4d. Such twinning structures have been often observed in previous MD simulation works in both the austenitic [30,33] and martensitic transitions [56]. The fcc stripes in the film mostly consisted of a single or double fcc atom layer(s), which are regarded as stacking faults produced by the high temperature, see the lower Figure 4c,d. In addition, considerable stresses were created in x and y directions (around 49 and −85 MPa) at the transition point. In the z direction, the stress was well relaxed by the free surface. This led to the buckled surface structure during/after the phase transition. Such a sudden stress increase at the transition points has been also reported in a study of phase transition in the Fe-C bulk system [30].

In films 1 and 2, the TBs mainly assisted the phase transition rather than the free surfaces. Nucleation in the bulk material or at the free surface, at least directly at the free surface, was not observed. Meiser and Urbassek [12] simulated a thin Fe slab (6.4 nm thick) with the (111)_bcc_ free surface and no austenitic phase transition could be observed during heating up to 2000 K. They attributed this to the absence of the (111)_bcc_ free surface in any transformation pathways. The ($11\bar{1}$)_bcc_ surfaces in films 1 and 2 may only provide free volumes for the coordinated atom movement rather than the nucleation sites. This should be the reason why the transition temperature difference between films 1 and 2 was only 50 K.

**Figure 5:**
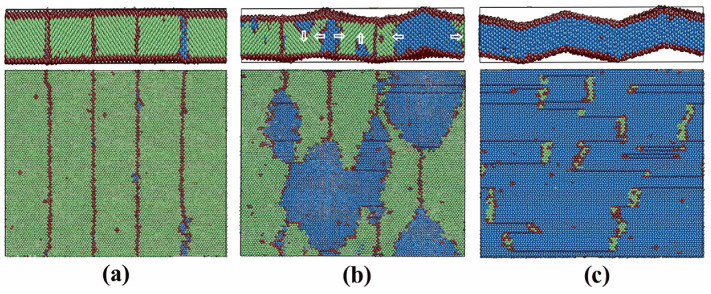
Snapshots of film 3 during the austenitic phase transition. Colors denote the local crystal structure as in Figure 1. The viewpoints are the same as in Figure 4. (**a**) The first nucleation at the TB and (**b**) nucleation at the free surface and the TB. The growth directions of the new phases are indicated by the white arrows and (**c**) final status after transition. Figure 5b has been revised.

Figure 5 shows the snapshots of film 3 during the austenitic phase transition. Analogous to films 1 and 2, the first nucleation starts at the TB (or the cross position between the TB and the free surface) with the further propagation along the TB plane, as shown in Figure 5a. After the full transition of the TB, the new phases grow in the transverse directions, as indicated by the horizontal white arrows in Figure 5b. Despite the TB nucleation, nucleation at the surface with afterward growth into the film can be also clearly observed. The vertical white arrows in Figure 5b indicate these growth directions. The hcp phase nucleated at the free surface had the same crystalline orientation as that nucleated at the TBs. The reason for the formation of hcp phase rather than fcc phase was discussed in film 2. After the full growth, the whole film transformed from its original bcc structure to an almost pure hcp crystallite, except some bcc patches and stacking faults (fcc strips in the film), as shown in Figure 5c. In this case, both the free surface and the TB acted as the nucleation sites, when the surface atom fraction was extremely high. Note that the thickness of film 3 was only 2.24 nm. The part assisted by the surface should be the reason for the obviously lower austenitic temperature of film 3 compared with films 1 and 2.

#### 3.2.2. Dependence on TB Fraction

**Figure 6:**
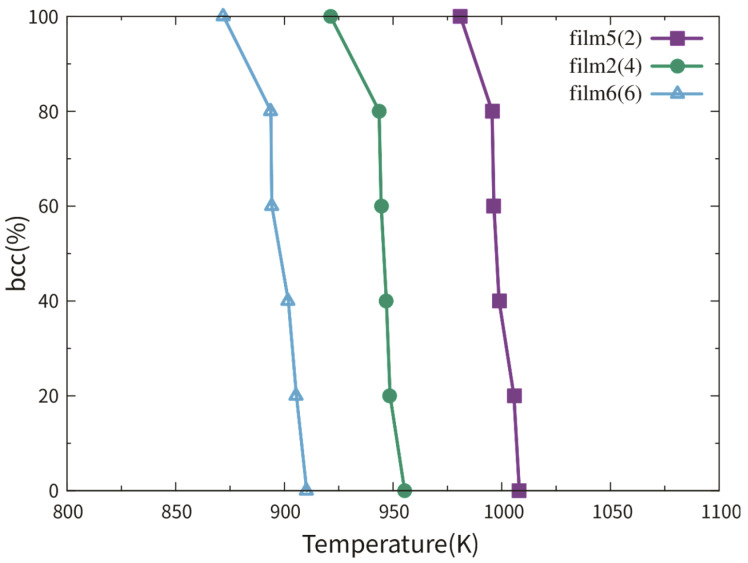
Fractional bcc phase content of films 5, 2 and 6 as a function of temperature. These three films have the same thickness and the numbers in the brackets indicate the number of TBs in the films.

Figure 6 shows the fractional bcc phase content as a function of temperature of films 5, 2 and 6. Film 4 is a single crystal thin film with the (111)_bcc_ surface. No austenitic phase transition could be observed during the heating from 50 to 2000 K. This observation can be supported by the work of Meiser and Urbassek [12]. So, the curve of film 4 is not shown in Figure 6. The austenitic transition temperatures of films 5, 2 and 6 were around 900 K, 950 K and 1000 K, respectively. In all three cases, the bcc fractions were smaller than 2% after the phase transition. Analogous to films 1 and 2, the phase transitions in films 5 and 6 were assisted by the TBs rather than the free surface. The new phases nucleate at the TBs (or in cross positions between the TB and free surface) and grow in the transverse directions until the whole film was totally transformed.

**Figure 7:**
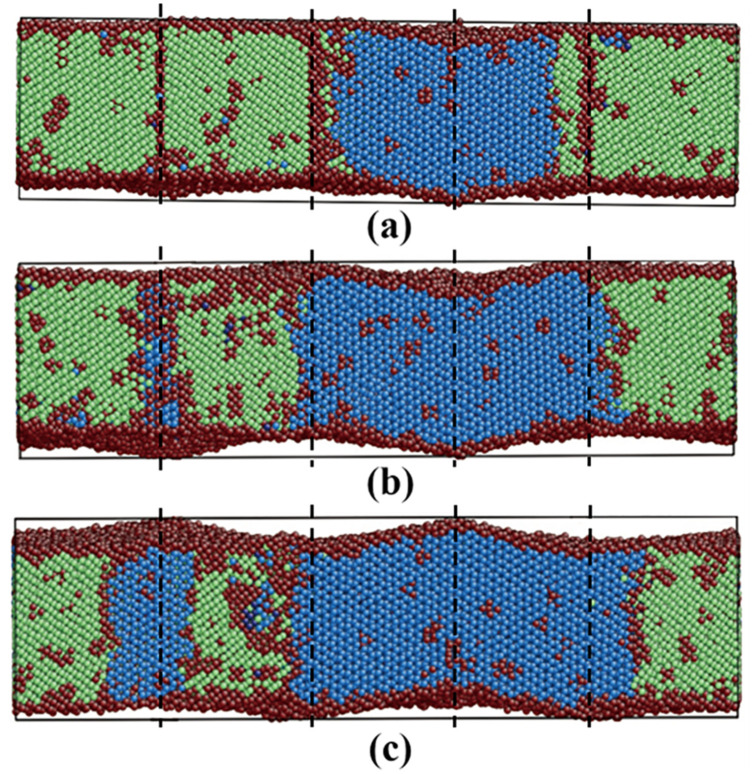
Snapshots of the ongoing austenitic transition in film 2. The plane as shown in the figure is the original ($1\bar{1}0$)_bcc_ plane. Colors denote the local crystalline structure as in Figure 1. The black dashed lines indicate the original TB positions. (**a**,**b**) The new hcp phase “devours” the neighbor TBs and new nucleation at the first TB and (**c**) growth of two close packed (cp) phases nucleated at different TBs.

It seems logical that the transition temperature decreased with the TB fraction increase. However, the highest (film 5) and lowest (film 6) transition temperature only had a difference of 100 K. This can be explained by the dynamics. The TB, at which the first nucleus appears, is random, since the temperature/pressure fluctuations in the film are not precisely controllable by the thermostat/barostate. Once the first nucleation took place, the new phase grew rapidly. Note that all the austenitic transitions in our study were completed within 50 ps. There were two possibilities after the first nucleation, as shown in Figure 7. (i) The new phase grows until it has contact with its neighbor TB, at which no nucleation takes place, and “devours” it, see the second and fourth TBs (from left to right) in Figure 7a,b. Dynamically, this “devouring” process was completed instantaneously and could not be captured by our highest simulation resolution of 0.5 ps. (ii) During the growth of the first nucleus, a new phase nucleates at its neighbor TB, see the first TB in Figure 7b. Both of them grow rapidly, see Figure 7c, and combine it with each other. Although a higher TB fraction offered more nucleation sites, the nucleation chances for each TB were exactly equivalent. Note that a high heating rate of 1 K/ps was performed in our simulations so that the thermodynamic condition (overheating) will be rapidly fulfilled for all the TBs. This equivalent nucleation chance for each TB and the rapid growth afterward led to the small difference between the transition temperatures.

#### 3.2.3. Pathway of the Austenitic Phase Transition

**Figure 8:**
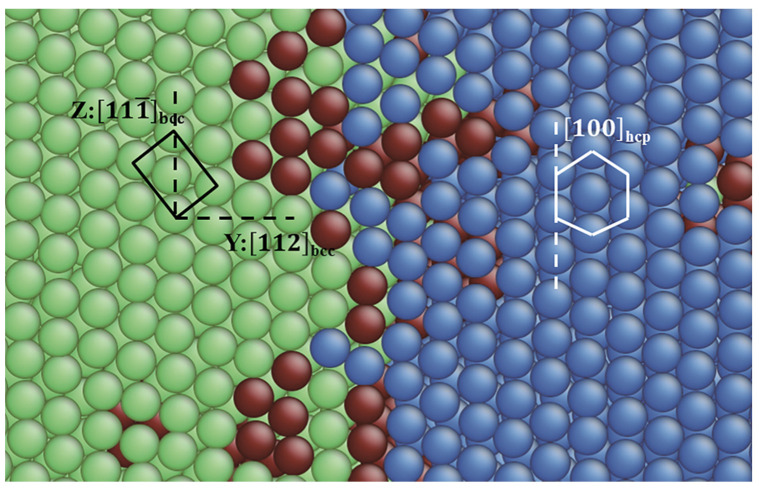
A zoomed transforming area of the (1$\bar{1}$0)_bcc_ plane containing a bcc/hcp phase boundary from film 5. Colors denote the local crystalline structure as in Figure 1. The black rectangle and the white hexagon indicate a unit cell on the (1$\bar{1}$0)_bcc_ plane and the transformed hexagonal structure on the (001)_hcp_ plane.

The bcc→hcp transitions in films 1–3 and 5–6 obey the same transition pathway. A zoomed snapshot from film 5 was taken as an example to illustrate the path. Figure 8 displays a transforming area of the original ($1\bar{1}0$)_bcc_ plane containing a bcc/hcp phase boundary. The y and z directions indicated by the black dashed lines correspond to the [112]_bcc_ and [$11\bar{1}$]_bcc_ directions. The [100]_hcp_ direction is indicated by the white dotted line. The transition follows the Burgers path [57] with the orientation relationship

(2) $$(110{)}_{\mathrm{bcc}}\;//\;(001{)}_{\mathrm{hcp}}\;\mathrm{and}\;[11\bar{1}{]}_{\mathrm{bcc}}\;//\;[100{]}_{\mathrm{hcp}},$$

This orientation relationship has been also found in strain induced bcc→hcp transitions [13,23]. Note that in the Burgers [57] path, the close packed plane and direction in the bcc structure are parallel to the close packed plane and direction in hcp structure. This is analogous to the Kurdjumov-Sachs (K–S) relationship [58] in the α–γ phase transition, which is often observed in pure Fe or low carbon steels. 

### 3.3. Martensitic Phase Transition

#### 3.3.1. Dependence on Film Thickness

**Figure 9:**
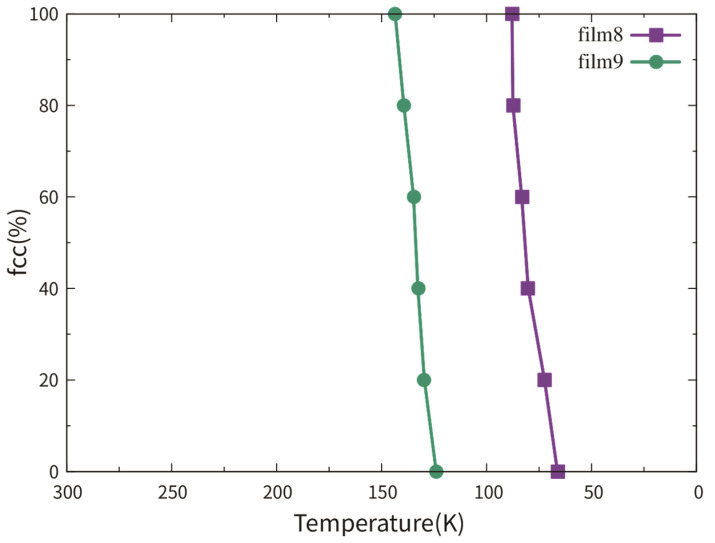
Fractional fcc phase content of films 8 and 9 as a function of temperature. Film 9 is as half thick as film 8.

Films 7–9 were investigated to study the influence of the film thickness on the martensitic phase transition. The detailed information of films 7–9 is listed in Table 2. Figure 9 shows the fractional fcc phase content of films 8 and 9 as a function of temperature. For film 7 with the biggest thickness of 8.87 nm, no martensitic phase transition can be observed during the cooling, despite the presence of the TBs. So, the curve of film 7 is not shown in Figure 9. In a previous simulation work using the Meyer-Entel potential [12], a very low martensitic transition temperature of 81 K was found for a 4 nm thick fcc single crystalline film with the (110) surface. In that work, the bcc phase nucleats homogeneously in the bulk rather than at the surface. The martensitic transition temperatures of film 8 and 9 were approximately 83 K and 134 K. In references [14,30] using the periodic boundaries in all three directions, much higher martensitic transition temperatures around 280 K were reported. Note that in those simulations, heating/cooling cycles were applied to the systems, i.e., the fcc phases at high temperatures were transformed from the bcc phases. Such fcc phases may contain many imperfections, in particular vacancies, which facilitate the martensitic phase transition, see detailed discussion about the heating/cooling rate in Section 2. Under the same conditions of the TB fraction and orientation, the transition temperature of film 9 was higher than that of film 8. This is logical because higher surface fraction provides more space for the new phase nucleation, so that the new phase can be formed more rapidly in thinner films. In films 8 and 9, the bcc phases nucleated both at the TBs and the surface, for details see the next paragraph. Note that the {111}_fcc_ TBE only amounted to −0.0073 J/m^2^, indicating that the TB atoms did not differ a lot from the bulk atoms energetically. The TBs provide structure fluctuation for the nucleation rather than energy fluctuation.

**Figure 10:**
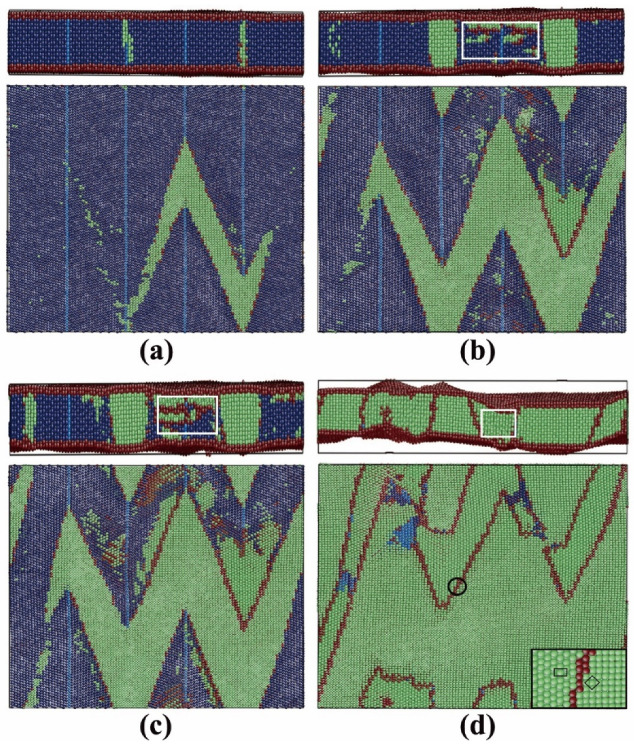
Snapshots of the ongoing martensitic phase transition in film 9. Colors denote the local crystal structure as in Figure 1. In a–d, the upper figures show the side view in the [11$\bar{2}$]_fcc_ direction (x) and the lower figures show the vertical views in the [$\bar{1}10$]_fcc_ direction (z). In the lower figures, the film is cut in the middle and the upper part is removed. (**a**) First nucleation at the TBs; (**b**) growth of the bcc phase nucleated at the TBs and new nucleation at the free surfaces; (**c**) growth of the bcc phases nucleated at the TBs and the free surfaces; and (d) final status after the martensitic transition.

In films 8 and 9, both the TB and the free surface attribute to the new phase nucleation and the transition processes were similar. Film 9 was taken as an example to illustrate the martensitic phase transition, as shown in Figure 10. Analogous to the austenitic transition, the first nucleation of the bcc phase started at the TBs (or intersection between the TB and the surface) with afterward growth along the TB planes, see Figure 10a. Then, new bcc phases nucleated at the free surfaces and grew into the film in the opposite surface normal direction, while the ones nucleated at the TBs propagated in transverse directions, see Figure 10b,c. The white rectangles in Figure 10b–d indicate a bcc phase nucleated at the free surface with afterward growth. The surface nucleation lagged by only 1 ps after the TB nucleation. Thus, simultaneous nucleation at different sources could be assumed. These two bcc phases had different crystalline orientations and were separated by the grain boundaries after the complete growth, as shown in Figure 10d. The zoomed subfigure in Figure 10d, which corresponds to the area indicated by the black circle, shows this orientation difference. The unit cells in the two bcc phases are indicated by the black square and rectangle. The orientation relationships of the interfaces between the fcc and the two types of bcc phases were equivalent in all the fcc films simulated, for detailed information see Section 3.3.3. In addition, the stress in the surface normal direction (z) was efficiently relaxed by the free surface, which caused the bulked surface during/after the martensitic phase transition, see the upper Figure 10c,d. Thus, it could be concluded that although the TBs also served as the nucleation sites (equivalent in films 8 and 9), the increased surface fraction (film 9) should be the reason for the martensitic temperature increase, as always observed in surface-dominated systems such as nanowires [7,8] or thin films [12].

#### 3.3.2. Dependence on the TB Fraction

**Figure 11:**
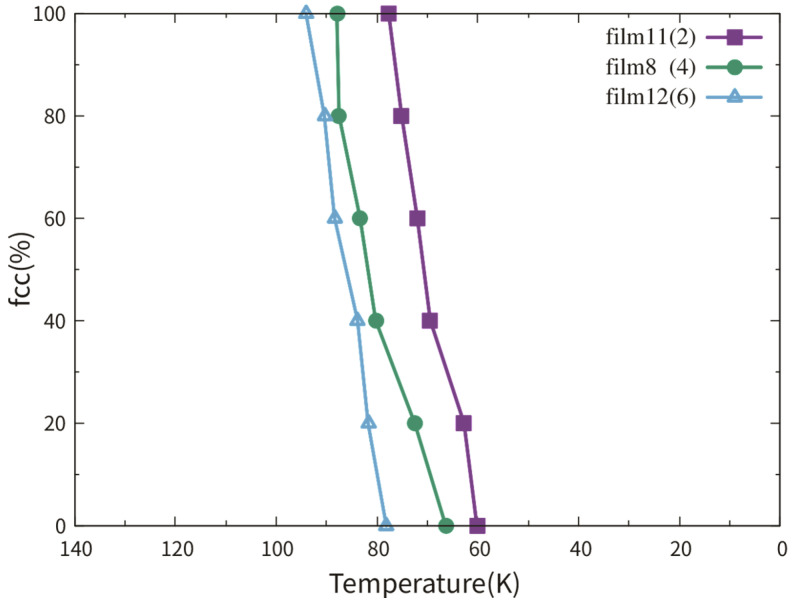
Fractional bcc phase content of films 11, 8 and 12 as a function of temperature. These three films have the same thickness and the numbers in the brackets indicate the number of TBs in the films.

Figure 11 shows the fractional fcc phase content of films 11, 8 and 12 as a function of temperature. No phase transition can be observed in film 10 with a single crystalline structure during cooling. This observation corresponds to the result in reference [12]. So, the curve of film 10 is not shown in Figure 11. The reason for the transition temperatures lower than 100 K was discussed in Section 2 and Section 3.3.1. The martensitic temperature did not show an increasing tendency with the TB fraction increase. Note that the difference between the martensitic temperatures of films 11, 8 and 12 was smaller than 20 K.

The transition processes of films 8, 9, 11 and 12 were analogous. The bcc phases with different crystalline orientations nucleated almost simultaneously at the free surfaces and the TBs. In film 11, the first nucleation at the free surface took place even 1 ps earlier than that at the TB. Polycrystalline bcc films were formed after the complete growth. The three films shown in Figure 11 had the same thickness and orientation, so the free surfaces contributed equivalently to the phase transitions. As mentioned in Section 3.2.2, each TB had the same opportunity to be the nucleation site, once the thermodynamic condition (supercooling) was fulfilled. Note that a high cooling rate of 0.33 K/ps was applied to the films. The TBs, at which no nucleation occurred, were rapidly “devoured” by the growing bcc phases. For all the fcc films simulated, the martensitic phase transitions were completed in a few tens of picoseconds. As a similar reason for the weak dependence of the austenitic transition temperature on the TB fraction, see Section 3.2.2, the TB fraction did not influent the martensitic temperature.

#### 3.3.3. Pathway of the Martensitic Phase Transition

**Figure 12:**
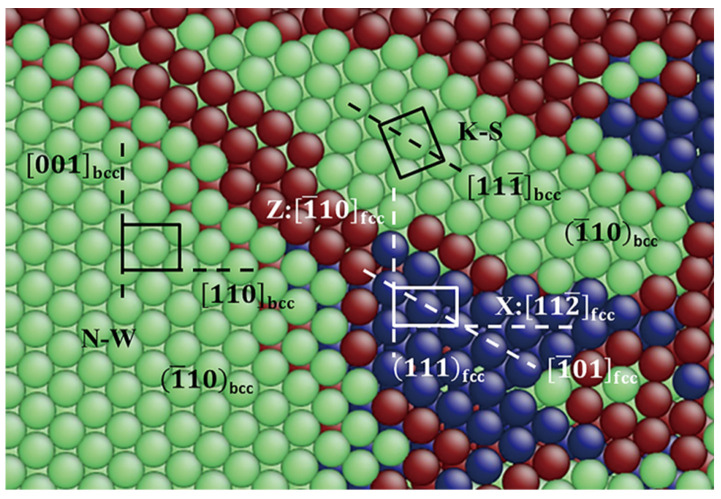
A zoomed snapshot of the ongoing martensitic phase transition in film 12. Colors denote the local crystalline structure as in Figure 1. The plane as shown in the figure is the original (111)_fcc_ plane and the transformed $(\bar{1}$10)_bcc_ plane. The red atoms at the top are surface atoms. The white rectangle indicates an fcc unit cell. The black rectangles on the left and right side indicate two unit cells in the bcc phases nucleated at the TB and the surface, respectively (this figure has been revised).

The bcc phases nucleated at the free surface and the TB constituted different crystalline relationships with the parent fcc phase. Such relationships were equivalent in all the fcc films studied. Figure 12 shows a zoomed snapshot of the ongoing martensitic transition in film 12, where the two types of bcc phases and the parent fcc phase coexisted. The plane, as shown in Figure 12, is the original (111)_fcc_ plane whose normal is the y-direction of the film. Note that the TBs were not visible on this plane. The (111)_fcc_ plane was transformed to the ($\bar{1}10)$_bcc_ plane, i.e., the close packed plane of the fcc phase was parallel to that of the bcc phase. Over 90% of the α/γ phase transitions in steel follow this relationship [59]. For the bcc phase nucleated at the TB (left), the [11$\bar{2}$]_fcc_ direction was transformed to the [110]_bcc_ direction and the [$\bar{1}10$]_fcc_ direction was transformed to the [001]_bcc_ direction. This corresponds to the Nishiyama–Wassermann (N–W) [60] orientation relationship,

(3) $$(111){}_{\mathrm{fcc}}\;//\;\left(\bar{1}10\right){}_{\mathrm{bcc}}\;\text{and}\;[11\bar{2}]{}_{\mathrm{fcc}}\;//\;[110]{}_{\mathrm{bcc}}$$

For the bcc phase nucleated at the surface (right), it can be observed that the close packed directions of the bcc and fcc phases, namely, the [$\bar{1}01$]_fcc_ and the [$11\bar{1}$]_bcc_ directions were parallel to each other. This is in accordance with the K–S [58] orientation relationship.

(4) $$(111){}_{\mathrm{fcc}}//\;\left(\bar{1}10\right){}_{\mathrm{bcc}}\mathrm{and}\;[\bar{1}01]{}_{\mathrm{fcc}}//\;[11\bar{1}{]}_{\mathrm{bcc}},$$

Karewar et al. [14] also found the N–W relationship in the martensitic transition initiated from the TBs in bulk Fe, which can be solidly supported by our result. Meiser and Urbassek [12] simulated the martensitic phase transition of a single crystalline Fe thin film with (110)_fcc_ free surface and reported the K–S relationship. However, in their work, the bcc phase nucleated homogeneously in the bulk material rather than the (110)_fcc_ free surface. It is possible that the martensitic transition follows two different pathways in one system due to the different local structures (TB or free surface) as nucleation sites. Yang et al. [3] investigated the deformation included martensitic transformation (DIMT) in 304 austenitic stainless steel treated with surface mechanical attrition treatment (SMAT) [61]. The observations of the authors verified for the first time the 50-year-old Bogers–Burgers–Olson–Cohen (BBOC) model [62,63,64,65], where steel undergoes an γ→ε→α’ transition. In their work, the Pitsch [66] and K–S orientation relationship co-survive in a martensitic particle embedded inside a single austenitic crystal, which affirms the possibility that in one system different orientation relationships coexist, like the K–S and N–W orientation relationships in our case. Note that the Pitsch orientation relationship was also found in a simulation work of martensitic phase transition of Fe thin slab with (100)_fcc_ surface using the Meyer-Entel potential [12]. In an experimental work of Shen et al. [4], the orientation relationships between the γ, ε and α’ phases during the martensitic phase transition were analyzed. It was found that the hcp (as an intermediate phase in the martensitic phase transition) to bcc obeys the Burgers path, which is in accordance with our observation during the bcc→hcp phase transition, see Figure 8. Furthermore, a K–S relationship between the fcc and bcc phases was also found by the authors, which partially corresponds to our observation (in our case both the K–S and N–W relationship). The viewpoint of the authors that the new phase preferentially nucleates at intersections of the TBs (in our case intersection between the TB and the free surface) is solidly supported by our observation, see Figure 4 and Figure 10. However, the intermediate hcp phase during the martensitic phase transition in references [3,4] is not observed in our simulation. This is due to the reality that it lacks the necessary plastic deformation to induce the martensitic fcc→hcp in our pure thermally induced phase transition. Finally, we may note the work of Johnston et al. [67], where the K–S orientation relationship was found in pure iron films on the Ni/W(110) substrate during the fcc→bcc structural change. This is in partial agreement with our simulation result.

### 3.4. Transition Temperature Hysteresis

The Meyer-Entel potential gives an equilibrium transition temperature of 550 ± 50 K. This equilibrium temperature, at which the free energies of the bcc and fcc phases are identical, was characterized via the method of metric scaling and suitable for the most available transition pathways [42]. The transition barrier is 7 meV/atom at this temperature [33]. In studies using the Meyer-Entel potential, inclusive of this work, the austenitic temperatures were always above the equilibrium temperature, and the martensitic temperatures were always below it [14,17,30]. In the phase transition induced by temperature changing, the finite heating/cooling rate should be the reason for this hysteresis as a result of the kinetic effect. In MD simulation, the much higher heating/cooling rate compared with that in reality makes this hysteresis effect even bigger. The transition temperature dependence on the heating/cooling rate has been discussed elsewhere [30]. We note that this is not a simulation artifact and such hysteresis also occurs in reality, for instance, homogeneous melting takes place above thermodynamic melting point. In the bulk, it is not easy to induce solid–solid phase transition via MD simulation. For instance, Meiser and Urbassek [12] simulated bulk Fe in the fcc structure with a cooling rate of 0.33 K/ps using the Meyer–Entel potential and no phase transition could be observed. Note that the starting point of their simulation was a perfect fcc crystal without any defects. Thus, an often used practice is to introduce a high percentage of vacancies into the system [28], even it is unphysical. In this work, the factors that facilitate the phase transition are the free surface and the TB. We mainly focus on the effect of the free surface and TB on the phase transition and their qualitative influences on the transition temperature rather than quantitative effects.

## 4. Conclusions

Using classical atomistic simulation, the austenitic and martensitic phase transitions in different thick Fe thin films containing various fractions of TB were studied. The energies of the {112}_bcc_ and {111}_fcc_ TBs were calculated and compared with the literature values. Both the influences of film thickness and TB fraction on the austenitic/martensitic phase transition were investigated and the transition pathways were analyzed. The main results are concluded as the follows.

1. Both the (112)_bcc_ and (111)_fcc_ TBs were stable in the thin films with the perpendicular orientation relationship with the free surface. The TBE was 0.176 J/m^2^ (−0.0073 J/m^2^) for the bcc (fcc) TB. The difference between these two energies could be attributed to the different atomistic arrangement at the TBs.

2. In the austenitic transition, the transition temperature shows weak dependence on the film thickness, when the free surface did not participate in the nucleation (films 1 and 2). Nucleating the new phase at the free surface (film 3), an obvious transition temperature decrease could be observed. The TB fraction shows only slight influence on the austenitic temperature (films 2, 5 and 6). The reasons for it are the equivalent nucleation chances for each TB caused by the high heating rate and the rapid growth afterward. The bcc→hcp phase transition obeys the Burgers pathway, which is analog to the K–S relation in the α/γ transition.

3. In the martensitic phase transition, the transition temperature increased with decreased film thickness (films 8 and 9). This is due to the reality that besides TBs, the free surfaces also assist the phase transition as nucleation sites. Dependence of the transition temperature on the TB fraction (films 8, 11 and 12) was not found due to the analogous reason in the austenitic phase transition. The whole martensitic transition process can be divided into three steps. (i) Simultaneous nucleation of the bcc phases at the TB and the free surface. Note that the two bcc phases exhibit different crystalline orientations. (ii) Growth of the bcc phases (iii) A bcc polycrystalline film after the complete transition. Both K–S and N–W relationships were observed in our transforming thin films.

## Figures and Tables

**Table 1:** Detailed information for the bcc (face-center-cubic) films 1–6. X, y and z indicate the orientations in each direction. Z gives the orientation of surface normal. Δx, Δy and Δz are the thicknesses in each direction. T and N denote the twin boundary (TB) number contained in the film and total atom number, respectively. See Figure 1a,b for a sketch of film 2.

Film	x	y	z	Δx (Å)	Δy (Å)	Δz (Å)	T	*N*
1	[$1\bar{1}$0]	[112]	[$11\bar{1}$]	202.9	210.9	89.4	4	324000
2	[$1\bar{1}$0]	[112]	[$11\bar{1}$]	202.9	210.9	44.7	4	162000
3	[$1\bar{1}$0]	[112]	[$11\bar{1}$]	202.9	210.9	22.4	4	81000
4	[$1\bar{1}$0]	[112]	[$11\bar{1}$]	202.9	210.9	44.7	0	162000
5	[$1\bar{1}$0]	[112]	[$11\bar{1}$]	202.9	210.9	44.7	2	162000
6	[$1\bar{1}$0]	[112]	[$11\bar{1}$]	202.9	210.9	44.7	6	162000

**Table 2:** Detailed information for the fcc films 7–12. The letters in the first line indicate the same as in Table 1. See Figure 1c,d for a sketch of film 8.

Film	x	y	z	Δx (Å)	Δy (Å)	Δz (Å)	T	*N*
7	[11$\bar{2}$]	[111]	[$\bar{1}10$]	216.9	210.9	88.7	4	323136
8	[11$\bar{2}$]	[111]	[$\bar{1}10$]	216.9	210.9	44.4	4	161568
9	[11$\bar{2}$]	[111]	[$\bar{1}10$]	216.9	210.9	20.9	4	80640
10	[11$\bar{2}$]	[111]	[$\bar{1}10$]	216.9	210.9	44.4	0	161568
11	[11$\bar{2}$]	[111]	[$\bar{1}10$]	216.9	210.9	44.4	2	161568
12	[11$\bar{2}$]	[111]	[$\bar{1}10$]	216.9	210.9	44.4	6	161568

## Appendix A

Six interatomic potentials in EAM form for Fe were analyzed by Engin et al. [27]. Among them, only the Meyer-Entel potential [28] is able to describe both the austenitic and martensitic phase transitions. This potential predicts that the free energy curves of bcc and fcc phases intersect at a temperature of 550 ± 50 K, while the other potentials describe such a tendency: free energies of the bcc phase are always lower than the fcc phase between 100 and 1700 K. Detailed information about the EAM potentials see reference [27]. Other two available potentials in the non-EAM form, which can also describe both the austenitic and martensitic phase transitions, are the Müller potential [44] and the Lee potential [39]. The performances of these two potentials and the Meyer–Entel potential are briefly summarized in Table A1.

**Table A1:** Brief information of the interatomic potentials for Fe that can describe both the austenitic and martensitic phase transitions. T denotes the equilibrium α/γ transition temperature. The Lee potential gives two transition temperatures since the authors designed two potentials with respect to the temperature and the pressure. The column “SFE” denotes the stacking fault energy of the fcc phase along the {111} <112> slip system. The column “finite-size system” indicates whether the potential is suitable to describe the nanoscaled system. The column “phase transition” indicates whether austenitic/martensitic phase transitions can be achieved by using the potential in the present work. Detailed information of the basic material parameters described by these potentials see references [27,39,44].

Name	Type	T (K)	SFE (mJ/m^2^)	Finite-Size System	Phase Transition	Calculation Speed
Meyer–Entel	EAM	550 ± 50 [27]	−54 [14]	Yes	Yes	fast
Müller	Bond-order	1030 [44]	-	No [44]	No	low
Lee	MEAM	1050;1075 [39]	37.5 [14]	-	-	-
